# N6AMT1 is a novel potential diagnostic, prognostic and immunotherapy response biomarker in pan-cancer

**DOI:** 10.18632/aging.204868

**Published:** 2023-07-12

**Authors:** Mingqi Wang, Jiajie Zhu, Yingquan Ye, Ping Li, Weijie Sun, Mei Zhang

**Affiliations:** 1 Department of Chinese Integrative Medicine Oncology, The First Affiliated Hospital of Anhui Medical University, Shushan, Hefei 230022, Anhui, China; 2 Department of Integrated Traditional Chinese and Western Medicine, Anhui Medical University, Shushan, Hefei 230022, Anhui, China; 3 Department of Gastroenterology, Tongde Hospital of Zhejiang Province, Xihu, Hangzhou 310012, Zhejiang, China; 4 Department of Infectious Diseases, The First Affiliated Hospital of Anhui Medical University, Shushan, Hefei 230022, Anhui, China

**Keywords:** N6AMT1, prognosis, pan-cancer, diagnosis, immunotherapy

## Abstract

Background: The N-6-adenine-specific DNA methyltransferase 1 (N6AMT1) is the only writer responsible for DNA 6mA modifications. At present, its role in cancer is still unclear, and further systematic pan-cancer analysis is needed to explore its value in diagnosis, prognosis and immunological function.

Methods: The subcellular localization of N6AMT1 was explored by UniProt and HPA database. The expression data and prognosis data of N6AMT1 were downloaded from the UCSC (cohort: TCGA pan-cancer), and the diagnostic and prognostic value of N6AMT1 in pan-cancer was explored. The value of N6AMT1-guided immunotherapy was explored through three cohorts (GSE168204, GSE67501 and IMvigor210 cohort). The correlation between N6AMT1 expression and tumor immune microenvironment was explored using CIBERSORT and ESTIMATE calculation methods, combined with TISIDB database. The biological role of N6AMT1 in specific tumors was explored by GSEA method. Finally, we explored chemicals affecting N6AMT1 expression through the CTD.

Results: N6AMT1 is mainly localized in the nucleus and differentially expressed in 9 cancer types. In addition, N6AMT1 showed early diagnostic value in 7 cancers and showed potential prognostic value in multiple cancer types. We also demonstrated that N6AMT1 expression was significantly associated with immunomodulator-related molecules, infiltration of lymphocyte subsets, and biomarkers of immunotherapy response. Furthermore, we show that N6AMT1 is differentially expressed in the immunotherapy cohort. Finally, we explored 43 chemicals that can affect N6AMT1 expression.

Conclusions: N6AMT1 has shown excellent diagnostic and prognostic capabilities in a variety of cancers, and it may reshape the tumor microenvironment and contribute to the ability to predict response to immunotherapy.

## INTRODUCTION

Cancer is currently the leading cause of death worldwide, killing more than 10 million people each year [1]. The main methods for cancer treatment are surgery, radiation, chemotherapy, and immunotherapy. However, although these methods greatly prolong the survival time of patients, some patients still have a poor prognosis. Therefore, an in-depth understanding of the molecular mechanisms underlying the occurrence and development of cancer will help us to find more accurate prognostic biomarkers and therapeutic targets, thereby providing new approaches to the diagnosis and treatment of cancer.

Recent research has shown that abnormal epigenetic modifications (DNA methylation, histone modifications, etc.) are closely related to the occurrence of cancer [2]. Importantly, such epigenetic modifications are tunable; thus, targeting epigenetic modifications represents a promising therapeutic approach for cancer. With the development of deep sequencing, the newly discovered epigenetic mark DNA N6-methyl-2’-deoxyadenosine (6mA) methylation has been shown to be widespread in the human genome [3]. Dysregulation of DNA 6mA modification is associated with embryogenesis [4], atherosclerosis [5], hypertension [6], chronic kidney disease [7], and cancer [3, 8, 9]. The N-6-adenine-specific DNA methyltransferase 1 (N6AMT1), a putative methyltransferase, was the first writer identified to be responsible for DNA 6mA modification [3]. Therefore, we speculate that N6AMT1 may have a crucial role in the occurrence and development of cancer. Studies have shown that abnormal expression of N6AMT1 can affect the progression of triple-negative breast cancer and affect drug resistance [10, 11]. In addition, increased expression of N6AMT1 significantly increased the proliferation and migration of HCC and tongue squamous cell carcinoma [12, 13]. However, its specific mechanism of action and its role in other cancers have not yet been studied. Given the limited reports on the role of N6AMT1 in cancer, it is difficult to draw any conclusions at this time. Therefore, a comprehensive analysis of N6AMT1 in different cancer types is required.

In this study, we determined the differential expression of N6AMT1 in pan-cancer and screened cancer types with diagnostic and prognostic values. Furthermore, this study explored the correlation of N6AMT1 expression with immunomodulators, lymphocyte subset infiltration, and immunotherapy biomarkers, and assessed the potential value of N6AMT1 in immunotherapy in different cancer types. Finally, we explored the chemicals that affect N6AMT1 expression. N6AMT1 was found to be a predictor of diagnosis, prognosis and immunotherapy response in multiple cancers. This study may broaden the clinical application of N6AMT1 in various cancers.

## MATERIALS AND METHODS

### Data collection

Transcriptomic data and clinical profiles of 33 tumors from The Cancer Genome Atlas (TCGA) were obtained from the University of California Santa Cruz (UCSC) Xena resource (cohort: TCGA pan-cancer) (https://xena.ucsc.edu/). In addition, somatic mutation data were obtained from TCGA (https://portal.gdc.cancer.gov/). Three immunotherapy response cohorts were from the public Gene Expression Omnibus database (https://www.ncbi.nlm.nih.gov/geo/). A cohort from the IMvigor210 trial of atezolizumab-treated advanced urothelial carcinoma [14]; a cohort of metastatic melanoma patients treated with anti-PD1 (GSE168204); and nivolumab-treated human renal cell carcinoma samples (GSE67501). Generally, the indicators used to assess immunotherapy efficacy are progressive disease (PD), stable disease (SD), partial response (PR), and complete response (CR). In this study, PD and SD patients were classified as non-responders, and CR and PR patients were classified as responders.

### Subcellular localization analysis of N6AMT1

The UniProt database (https://www.uniprot.org/) includes all known protein sequences with a complete functional annotation compendium [15, 16]. The Human Protein Atlas (HPA, https://www.proteinatlas.org/) is a free open database containing various tissue immunohistochemical (IHC) images and various cell immunofluorescence (IF) images [17]. We analyzed the subcellular localization of N6AMT1 using the UniProt and HPA databases. IF pictures of N6AMT1 cell sublocalization in the human osteosarcoma cell line U-2 OS and human squamous cell carcinoma cell line A-431 were obtained from HPA. In addition, the IHC images of N6AMT1 protein expression in 7 normal tissues and corresponding tumor tissues were obtained through the HPA database.

### Clinical relevance of N6AMT1 expression and pan-cancer

First, we converted the transcriptome data (log2(FPKM+1)) acquired from UCSC Xena into a transcriptome data form (FPKM), consistent with the TCGA data. The limma package was used to analyze whether there was a difference in N6AMT1 expression between the tumor group and the normal group. Correlations between N6AMT1 expression and three clinical parameters (age, sex, and tumor stage) were also investigated.

### Analysis of the diagnostic value of N6AMT1

To evaluate the diagnostic accuracy of N6AMT1 in pan-cancer, we performed sensitivity- and specificity-based ROC curve analysis using the “Proc” package. The area under the curve (AUC) ranged from 0.5 - 1.0 [18]. Different AUC values represent different diagnostic values: no diagnostic value (AUC = 0.5), low diagnostic value (AUC: 0.5 - 0.7), relative diagnostic accuracy (AUC: 0.7 - 0.9), high diagnostic value (AUC: 0.9 -1.0), perfect diagnosis (AUC = 1.0).

### Analysis of the prognostic value of N6AMT1

We determined the prognostic value of N6AMT1 in pan-cancer using the survival and survminer packages based on several prognostic indicators: overall survival (OS), disease-free survival (DFS), disease-specific survival (DSS) and progression-free survival (PFS). For cancer types where N6AMT1 expression affects prognosis, we further supplemented Kaplan-Meier survival curve analysis. p < 0.05 was considered to indicate statistical significance.

### Gene set enrichment analysis (GSEA)

GSEA is a conventional tool for analyzing different groups based on gene expression data to provide insights of biological significance [19]. Gene Ontology (GO) gene set “c5.go.v7.4.symbols.gmt” and signal pathway gene set “c2.cp.kegg.v7.4.symbols.gmt” are obtained from GSEA website (https://www.gsea-msigdb.org/gsea/index.jsp). The samples were divided into high and low expression groups according to the median N6AMT1 expression value, and then GSEA functional analysis was performed using the “limma”, “enrichplot”, “clusterProfiler” and “org.Hs.eg.db” packages. Finally, the 5 most significantly correlated GO and signaling pathways are shown.

### Association of N6AMT1 expression with immune-related factors

Estimation of STromal and Immune cells in MAlignant Tumours using Expression data (ESTIMATE) is an algorithm based on single-sample gene set enrichment analysis (ssGSEA) that estimates the extent of stromal and immune cells in the tumor microenvironment (TME) using tumor expression data [20]. Thus, we obtained Immunescore and Stromalscore for each sample. CIBERSORT is an emerging deconvolution method for characterizing the composition of 22 immune cells based on tumor gene expression data [21]. Therefore, we used the CIBERSORT algorithm to analyze the proportion of 22 infiltrating lymphocyte subsets in the sample (perm = 1000, p < 0.05). TISIDB (http://cis.hku.hk/TISIDB/index.php) is a user-friendly portal with multi-type data resources integrating tumors and the immune system [22]. We explored the association between N6AMT1 and immunomodulators on a pan-cancer basis using the TISIDB website. The immunomodulators comprised 24 immunoinhibitors, 45 immunostimulators, and 21 major histocompatibility complex (MHC) molecules. We analyzed the potential associations of N6AMT1 with programmed cell death 1 ligand 1 (PDL1) expression, tumor mutational burden (TMB), microsatellite instability (MSI), and mismatch repair (MMR), which previous studies have identified as potential biomarkers for predicting good response to tumor immunotherapy [23]. TMB represents the number of nonsynonymous mutations per megabase in somatic cells [24]. MSI scores were derived from data from previously published studies [25].

### Interaction of N6AMT1 with chemicals

The Comparative Toxicogenomics Database (CTD; http://ctdbase.org/) is a publicly available large database linking toxicological information on chemicals, genes, phenotypes, diseases and exposures in understanding health [26]. We explored interacting chemicals with N6AMT1 using the CTD database.

### Cell culture, RNA extraction and quantitative real-timePCR (qRT-PCR)

All HCC cell lines HEPG2, BEL7402, HCCLM3 and normal liver cell line LO2 were donated by Dr. Dai [27]. Cell culture, RNA extraction, and qRT-PCR were performed as in previous studies [28]. The primer sequences involved in this study are as follows. β-actin primer forward sequences: CACCATTGGCAATGAGCGGTTC; β-actin primer Reverse sequences: AGGTCTTTGCGGATGTCCACGT. N6AMT1 primer forward sequences: GGCTTGCTACCAAGATTGACCG; N6AMT1 primer Reverse sequences: CCAAGCTGCCTCTATTCCGTGA.

### Statistical analysis

The TMB is obtained by processing the PERL programming language (version 5.32.1). All statistical analyses were performed using R software (version 4.1.0). Analyses of differential N6AMT1 gene expression were performed using Wilcoxon tests. Correlation analyses were performed using the Spearman correlation coefficient. p < 0.05 was considered to indicate a statistically significant difference.

## RESULTS

### Subcellular localization of N6AMT1 protein

**Figure 1:**
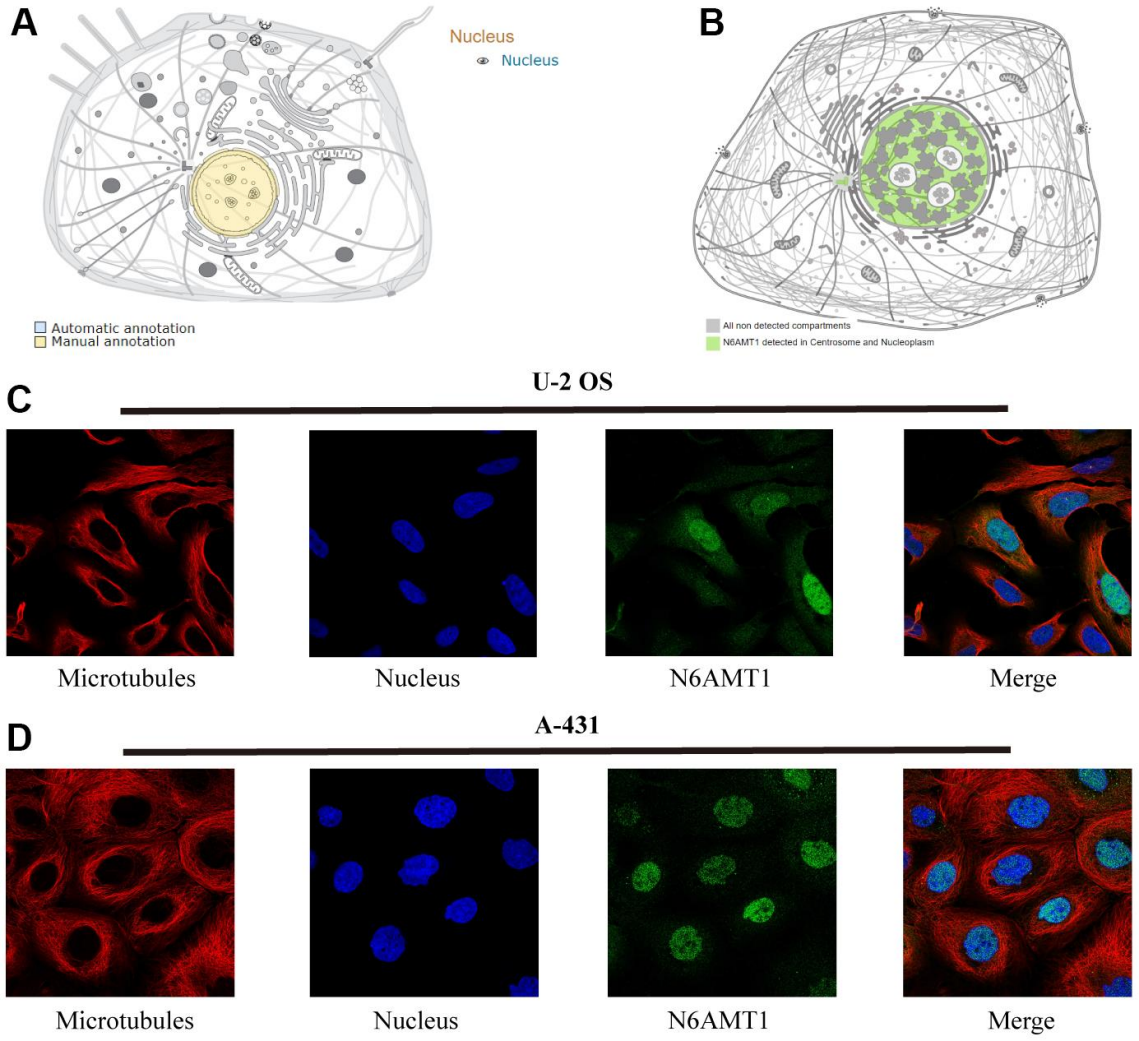
**Subcellular localization of N6AMT1 protein.** Annotations of N6AMT1 protein in the UniProt (**A**) and HPA (**B**) databases. Immunofluorescence images showing intracellular localization of N6AMT1 in U2-OS (**C**) and A-431 (**D**) cells.

N6AMT1 is a methylase that performs DNA 6mA modification. We first checked the intracellular localization of N6AMT1 protein in the UniProt and HPA databases; the results showed that N6AMT1 protein was mainly distributed in the nucleus (Figure 1A, 1B). Furthermore, IF results showed that N6AMT1 was mainly localized in the nucleus in U-2 OS (Figure 1C) and A-431 cells (Figure 1D). This provides a physical basis for N6AMT1 to perform DNA 6mA modification.

### Expression of N6AMT1 in 33 cancers

**Figure 2:**
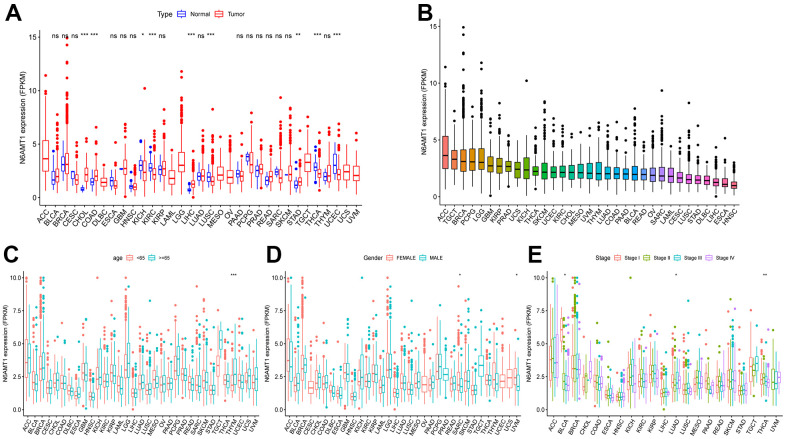
**Differential expression and clinical relevance of N6AMT1 in 33 tumor types.** (**A**) Differential expression of N6AMT1 in pan-cancer. (**B**) N6AMT1 expression in pan-cancer order from high to low. Correlations of N6AMT1 expression with patient age (**C**), gender (**D**) and tumor stage (**E**). *: p < 0.05, **: p < 0.01, ***: p < 0.001. ns: No Significant.

**Figure 3:**
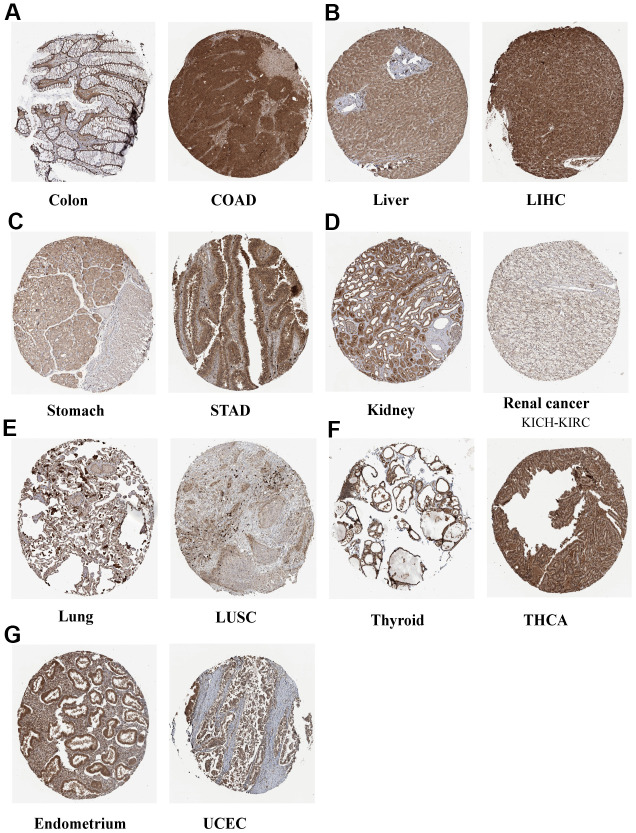
Representative IHC staining of N6AMT1 in eight normal (left) and tumor (right) tissues of the colon (**A**), liver (**B**), stomach (**C**), kidney (**D**), lung (**E**), thyroid (**F**) and endometrium (**G**).

The full names and abbreviations of the 33 cancers considered in this study are given in Table 1. Compared with normal tissues, N6AMT1 was differentially expressed in nine cancer types (CHOL, COAD, KICH, KIRC, LIHC, LUSC, STAD, THCA and UCEC) (Figure 2A); specifically, it showed significantly higher expression in CHOL, COAD, LIHC and STAD and low expression in KICH, KIRC, LUSC, THCA and UCEC. In addition, we analyzed and ranked the expression of N6AMT1 in tumor tissues and found that it had the highest expression levels in ACC and the lowest in HNSC (Figure 2B). Furthermore, we considered the relationship of N6AMT1 expression with clinical factors including age, gender and tumor stage; the results showed that N6AMT1 was differentially expressed in elderly patients aged ≥65 years in the THYM group (Figure 2C). Moreover, N6AMT1 expression was correlated with patient gender in the SARC and UVM groups (Figure 2D), with tumor stage in the BLCA, LUAD and THCA groups (Figure 2E). Furthermore, for the nine cancer types in which N6AMT1 was differentially expressed, we compared N6AMT1 protein expression levels between normal and tumor tissues using data obtained from the HPA database. The results showed that N6AMT1 was significantly overexpressed in COAD (Figure 3A), LIHC (Figure 3B) and STAD (Figure 3C) and significantly underexpressed in renal adenocarcinoma (KICH and KIRC) (Figure 3D), LUSC (Figure 3E), THCA (Figure 3F) and UCEC (Figure 3G). These results are consistent with the difference in mRNA expression.

**Table 1:** 33 types of human cancers employed in our research.

**Abbreviation**	**Full name**
ACC	Adrenocortical carcinoma
BLCA	Bladder urothelial carcinoma
BRCA	BRCA Breast invasive carcinoma
CESC	Cervical squamous cell carcinoma and endocervical adenocarcinoma
CHOL	Cholangiocarcinoma
COAD	Colon adenocarcinoma
DLBC	Diffuse large B-cell lymphoma
ESCA	Esophageal carcinoma
GBM	Glioblastoma multiforme
HNSC	Head and neck squamous cell carcinoma
KICH	Kidney chromophobe
KIRC	Kidney renal clear cell carcinoma
KIRP	Kidney renal papillary cell carcinoma
LAML	Acute myeloid leukemia
LGG	Brain lower grade glioma
LIHC	Liver hepatocellular carcinoma
LUAD	Lung adenocarcinoma
LUSC	Lung squamous cell carcinoma
MESO	Mesothelioma
OV	Ovarian serous cystadenocarcinoma
PAAD	Pancreatic adenocarcinoma
PCPG	Pheochromocytoma and paraganglioma
PRAD	Prostate adenocarcinoma
READ	Rectum adenocarcinoma
SARC	Sarcoma
SKCM	Skin cutaneous melanoma
STAD	Stomach adenocarcinoma
TGCT	Testicular germ cell tumors
THCA	Thyroid carcinoma
THYM	Thymoma
UCEC	Uterine corpus endometrial carcinoma
UCS	Uterine carcinosarcoma
UVM	Uveal melanoma

### Pan-cancer diagnostic value of N6AMT1

**Figure 4:**
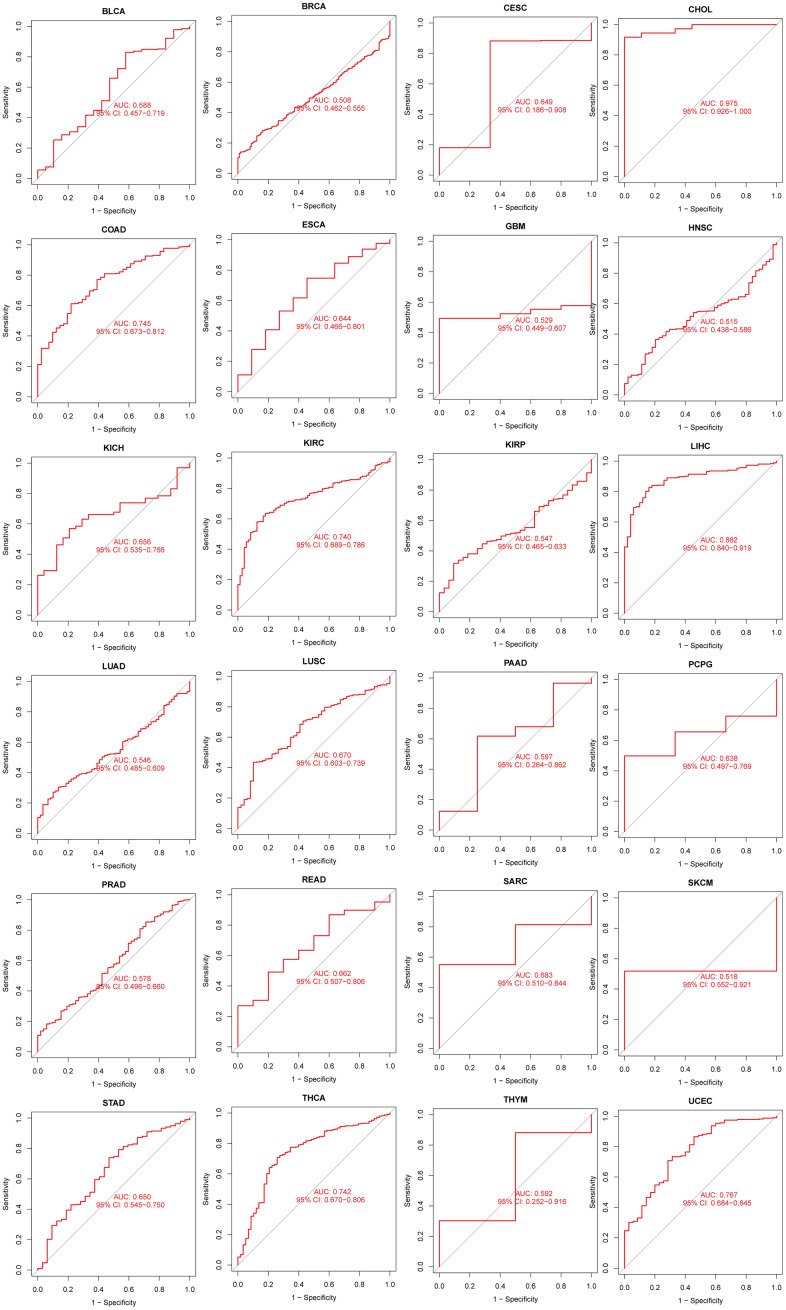
Analysis of the diagnostic value of N6AMT1 in pan-cancer.

We further evaluated the diagnostic ability of N6AMT1 in pan-cancer using ROC. Figure 4 shows that N6AMT1 exhibits high diagnostic accuracy in CHOL (AUC = 0.975). Relative diagnostic accuracy was demonstrated in COAD (AUC = 0.745), KIRC (AUC = 0.740), LIHC (AUC = 0.882), THCA (AUC = 0.742) and UCEC (AUC = 0.767). Lower diagnostic accuracy was demonstrated across 18 cancer types.

### Pan-cancer prognostic value of N6AMT1

**Figure 5:**
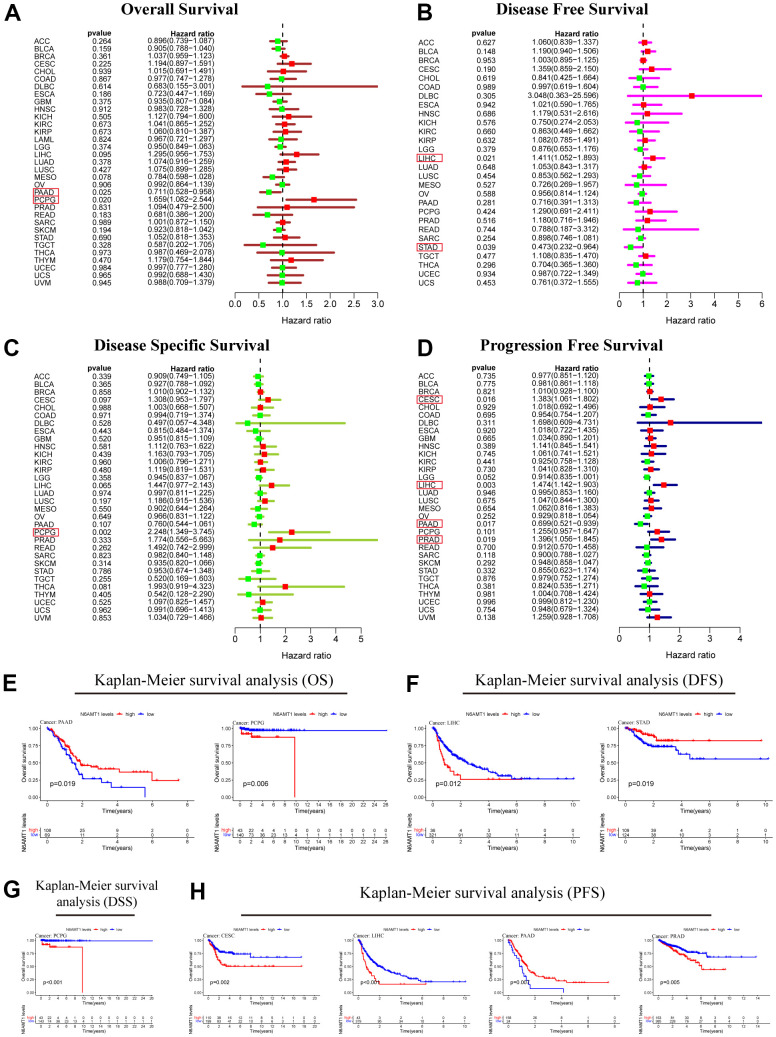
**Prognostic analysis of N6AMT1 in pan-cancer.** Forest plot showing the results of univariate Cox regression analysis of the correlations between N6AMT1 and OS (**A**), DFS (**B**), DSS (**C**) and PFS (**D**). (**E**–**H**) Kaplan-Meier survival curves of N6AMT1 in cancer types that affect cancer prognosis. p < 0.05 indicates statistical significance.

Next, we explored the relationship between N6AMT1 expression and patient prognosis in 33 cancer types. Univariate Cox regression analysis was performed; the results, illustrated by forest plots, showed that N6AMT1 expression was associated with OS in PAAD and PCPG patients (Figure 5A). Specifically, N6AMT1 was a protective factor for OS in PAAD patients (hazard ratio [HR] = 0.711, p = 0.025) and a risk factor for OS in PCPG patients (HR = 1.659, p = 0.020). However, there are other important clinical indicators that can reflect clinical benefit, such as DFS, DSS and PFS. Therefore, we further analyzed the correlations between N6AMT1 expression and these indicators. The results showed that N6AMT1 could affect DFS in LIHC and STAD (Figure 5B); specifically, N6AMT1 was a risk factor for DFS in LIHC (HR = 1.411, p = 0.021) and a protective factor for DFS in STAD (HR = 0.473, p = 0.039). Furthermore, N6AMT1 was a risk factor for DSS in PCPG (HR = 2.248, p = 0.002) (Figure 5C); a risk factor for PFS in CESC (HR = 1.383, p= 0.016), LIHC (HR = 1.474, p = 0.003) and PRAD (HR = 1.396, p = 0.019); and a protective factor in PAAD (HR = 0.699, p = 0.017) (Figure 5D). Finally, for cancer types where patient prognosis was affected by N6AMT1, we supplemented the analysis using Kaplan–Meier survival curves (Figure 5E–5H). In conclusion, our results suggest that N6AMT1 is closely related to patient prognosis, especially in PAAD, PCPG and LIHC.

### Correlations between N6AMT1 and the TME

**Figure 6:**
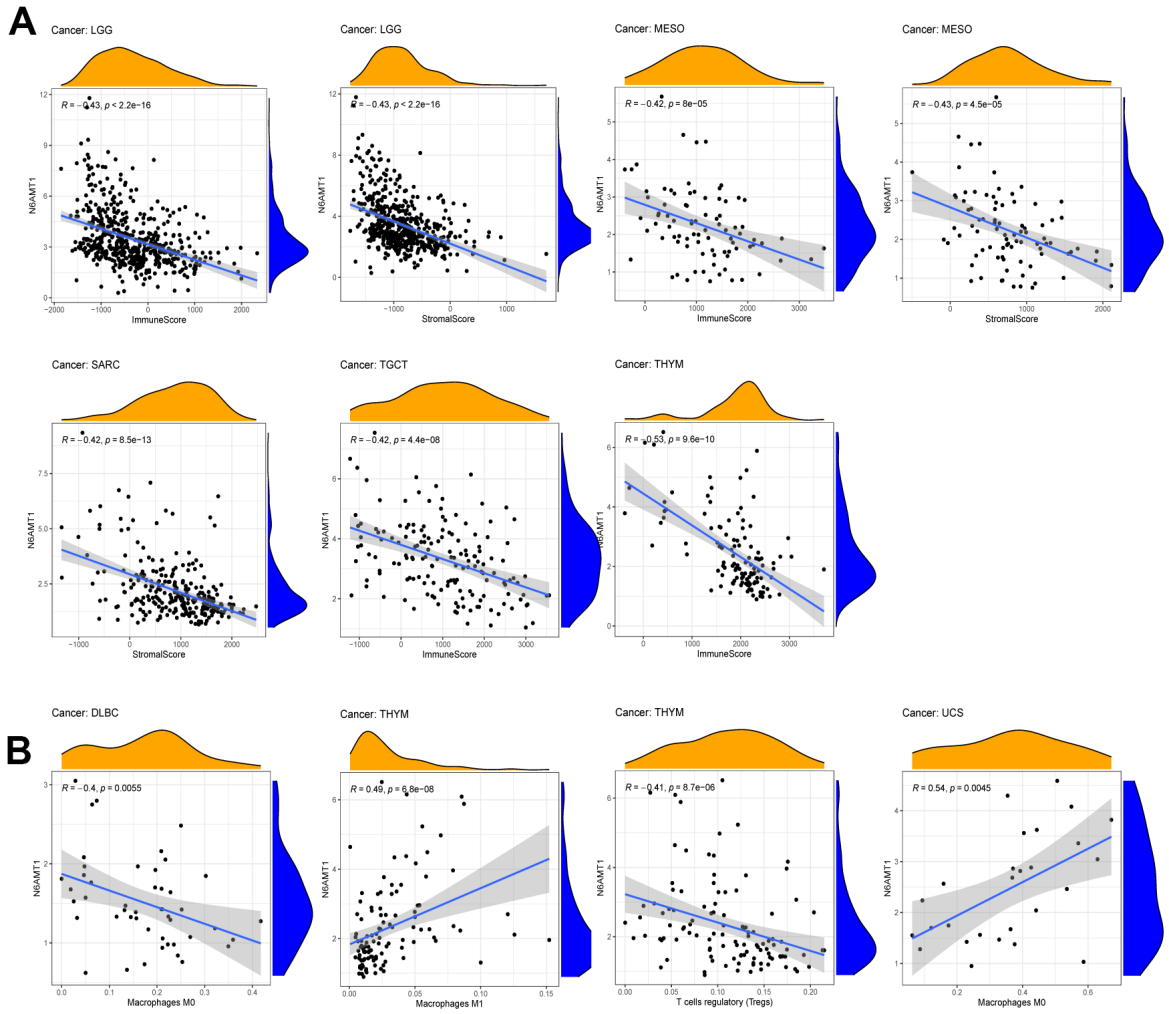
**Correlations between N6AMT1 expression and TME scores.** (**A**) Correlations between N6AMT1 and ESTIMATE scores including Immunescore and Stromalscore. (**B**) Correlation of N6AMT1 with infiltration of lymphocyte subsets.

To comprehensively explore the correlation between N6AMT1 and TME in pan-cancer, first, we analyzed the correlations between N6AMT1 expression and ESTIMATE scores, which included Immunescore and Stromalscore (filter criteria: |correlation coefficient| > 0.4, p < 0.01). The results showed that N6AMT1 expression was negatively correlated with Immunescore in LGG, MESO, TGCT and THYM and negatively correlated with Stromalscore in LGG, MSEO and SARC (Figure 6A). In addition, we explored the correlations between N6AMT1 expression and degree of immune cell infiltration using the CIBERSORT algorithm. In DLBC, N6AMT1 expression was negatively correlated with macrophages M0; in THYM, N6AMT1 expression was positively correlated with macrophages M1 and negatively correlated with regulatory T cells; in UCS, N6AMT1 expression was positively correlated with macrophages M0 (Figure 6B).

### Correlation of N6AMT1 expression with immunomodulators

**Figure 7:**
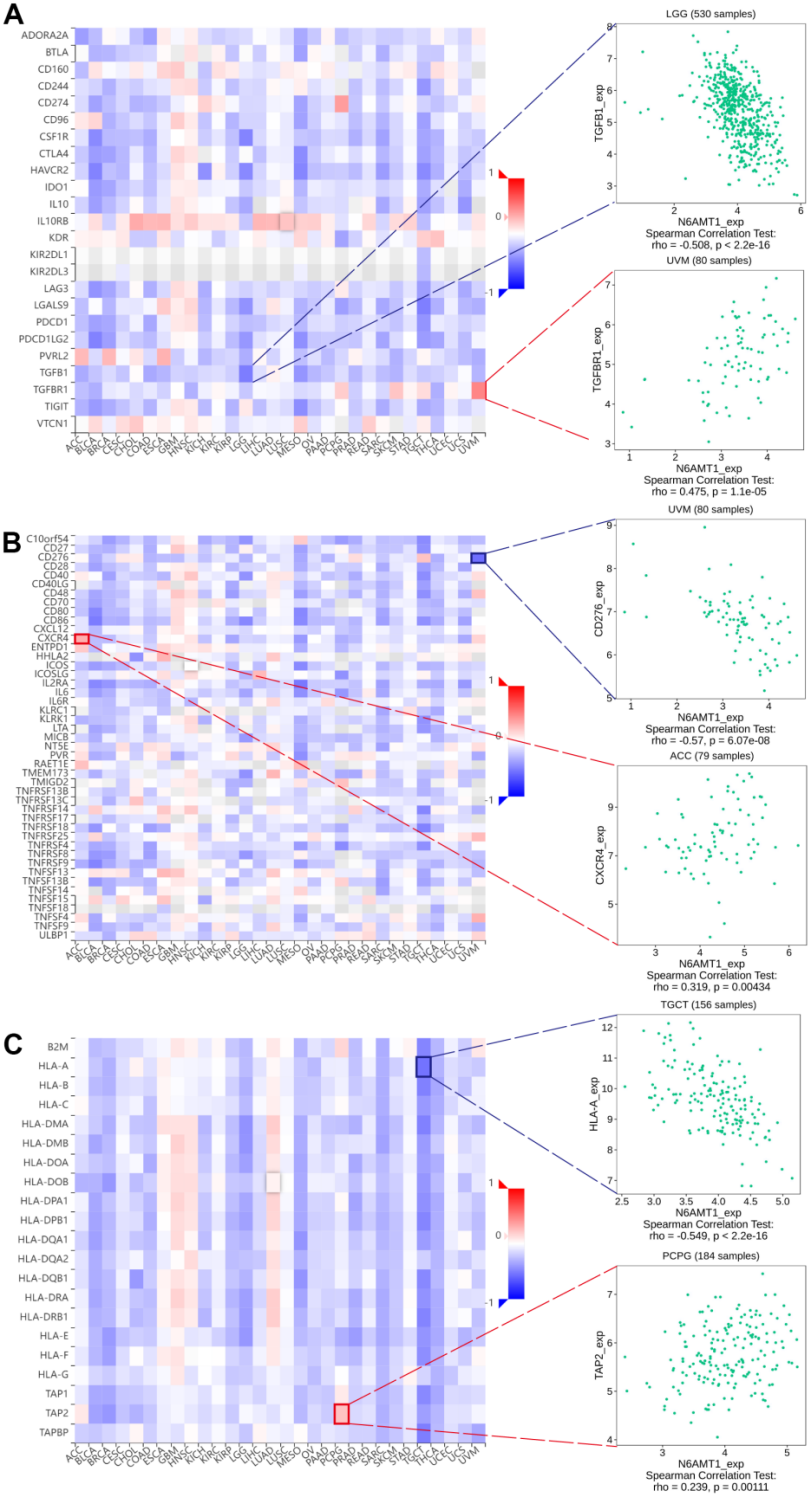
Correlations between N6AMT1 expression and immunomodulators: immunoinhibitors (**A**), immunostimulators (**B**) and MHC molecules (**C**). Red represents positive correlation and blue represents negative correlation. The most positively and negatively correlated cases are highlighted on the right.

**Figure 8:**
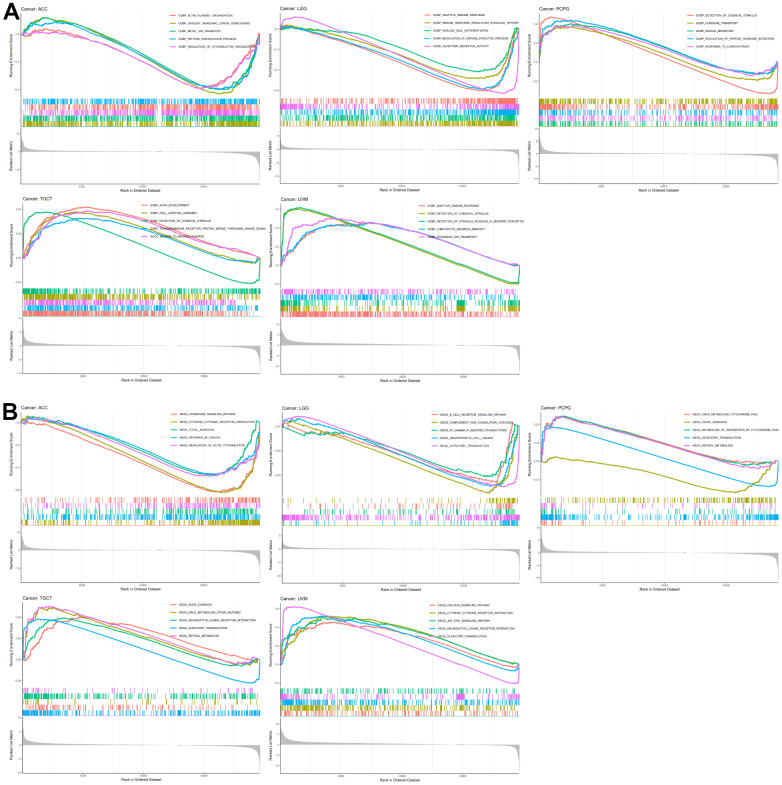
**N6AMT1-related GO and signal pathways.** (**A**) GO involving N6AMT1 in five tumor types as analyzed by GSEA. (**B**) Signal pathways involving N6AMT1 in five tumor types as analyzed by GSEA.

We queried TISIDB for correlations between N6AMT1 expression and immunomodulators, including immunoinhibitors, immunostimulators and MHC molecules. The correlation analysis of 24 immunoinhibitors showed that N6AMT1 expression was associated with most immunosuppressive agents on a pan-cancer basis: in UVM, N6AMT1 expression showed the strongest positive correlation with TGFBR1; and in LGG, N6AMT1 and TGFB1 expression showed the strongest negative correlation (Figure 7A). In addition, N6AMT1 expression had pan-cancer correlations with most of the 45 immunostimulators: in ACC, N6AMT1 expression showed the strongest positive correlation with CXCR4; in UVM, N6AMT1 expression showed the strongest negative correlation with CD276 (Figure 7B). Likewise, the correlations between 21 MHC molecules and N6AMT1 expression were analyzed. In PCPG, N6AMT1 expression showed the strongest positive correlation with TAP2; and in TGCT, N6AMT1 expression showed the strongest negative correlation with HLA-A (Figure 7C). Considering the strong correlations of N6AMT1 with ACC, LGG, PCPG, TGCT and UVM, GSEA was performed to investigate the GO and signal pathways involving N6AMT1 in these cancers. According to the results, the GO (Figure 8A) and signal pathways (Figure 8B) involving N6AMT1 varied widely in different cancers but were mostly related to tumor and immunity.

### Ability of N6AMT1 to predict response to immunotherapy

**Figure 9:**
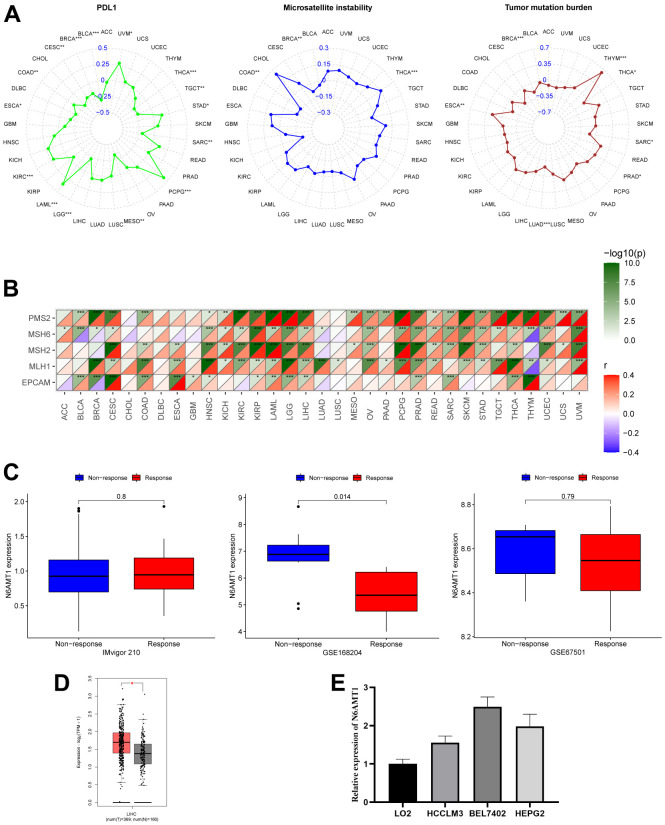
**Correlation of N6AMT1 expression with immunotherapy markers and immunotherapy response.** (**A**) Correlations of N6AMT1 expression with PDL1, MSI and TMB. (**B**) Correlations of N6AMT1 expression with MMR-related genes. (**C**) Differences in N6AMT1 expression between responder and non-responder groups in the three immunotherapy cohorts. (**D**) Differential analysis of mRNA expression of N6AMT1 in HCC in GEPIA2.0 database. (**E**) Expression levels of N6AMT1 in normal liver cell lines and HCC cell lines. *: p < 0.05, **: p < 0.01, ***: p < 0.001.

PDL1, TMB, MSI and MMR are currently considered as biomarkers to predict the response to immunotherapy [23]. Therefore, we explored the correlations between N6AMT1 expression and these biomarkers. PDL1 was positively correlated with N6AMT1 expression in KIRC, LAML, PCPG, STAD and UVM and negatively correlated in BLCA, BRCA, CESC, COAD, ESCA, LGG, MESO, SARC, TGCT and THCA. TMB was positively correlated with N6AMT1 expression in ESCA, PRAD and THYM and negatively correlated in BRCA, LUAD, SARC and THCA. MSI was positively correlated with N6AMT1 expression in THCA and negatively correlated with BRCA and COAD (Figure 9A). N6AMT1 expression was positively correlated with MMR-related genes (including MLH1, MSH2, MSH6, PMS2 and EPCAM) in most cancer types and was positively correlated with all MMR-related genes in CESC, HNSC, KIRP, LAML, LGG, LIHC, PRAD and THCA (Figure 9B). In addition, we analyzed three immunotherapy cohorts and found that in the GSE168204 cohort, N6AMT1 expression was significantly lower in the responder group than in the non-responder group (p = 0.014) (Figure 9C). By contrast, there was no significant difference in N6AMT1 expression between the responder and non-responder groups in the GSE67501 and IMvigor210 cohorts (Figure 9C).

### N6AMT1 expression verification in LIHC

First, we detected the expression of CTSA in liver cancer cell lines (BEL7402, HEPG2, HCCLM3) and normal liver cell lines (LO2), and the results showed that the expression level of CTSA in liver cancer cell lines was significantly higher than that in LO2 cells (Figure 9D). Secondly, we analyzed the expression of N6AMT1 in LIHC online through GEPIA2.0 (Merged TCGA and GTEx normal tissue expression data, http://gepia2.cancer-pku.cn/), and the results showed that N6AMT1 was significantly higher in cancer tissues than in adjacent normal tissues (Figure 9E).

### Interacting chemicals of N6AMT1

We explored N6AMT1-related chemicals using the CTD database. The results showed that a total of 45 chemicals were associated with N6AMT1 (Table 2). Among them, 19 chemicals can up-regulate N6AMT1 mRNA expression, and 24 chemicals can down-regulate N6AMT1 mRNA expression. In addition, there are two chemicals that can affect N6AMT1 mRNA expression, but the specific role is not clear.

**Table 2:** Interacting chemicals of N6AMT1 in CTD.

**Chemical name**	**Chemical ID**	**Interaction actions**	**Chemical name**	**Chemical ID**	**Interaction actions**
2,3,7,8-tetrachlorodibenzof-uran	C014211	Increases expression	(+)-JQ1 compound	C561695	Increases expression
2,6-dinitrotoluene	C023514	Increases expression	Methidathion	C005828	Increases expression
4-(5-benzo (1,3) dioxol-5-yl-4-pyridin-2-yl-1H-imidazol-2-yl) benzamide	C459179	Decreases expression	Methylmercuric chloride	C004925	Decreases expression
Abrine	C496492	Increases expression	Monomethylarso-nous acid	C406082	Increases expression
Acetamide	C030686	Decreases expression	Nickel	D009532	Decreases expression
Acetaminophen	D000082	Affects expression	Pentachlorophenol	D010416	Decreases expression
Aristolochic acid I	C000228	Decreases expression	Perfluoro-n-nonanoic acid	C101816	Increases expression
Benzo(a)pyrene	D001564	Increases expression	Pirinixic acid	C006253	Increases expression
Bisphenol A	C006780	Affects expression	Prochloraz	C045362	Increases expression
Cyclosporine	D016572	Decreases expression	Quercetin	D011794	Decreases expression
Cylindrospermopsin	C089595	Increases expression	Resorcinol	C031389	Decreases expression
Dicrotophos	C000944	Decreases expression	S-2-pentyl-4-pentynoic hydroxamic acid	C513635	Decreases expression
Dorsomorphin	C516138	Decreases expression	Soman	D012999	Decreases expression
Doxorubicin	D004317	Decreases expression	Sunitinib	D000077210	Increases expression
Endosulfan	D004726	Increases expression	Tetrachlorodiben-zodioxin	D013749	Increases expression
Ethinyl estradiol	D004997	Increases expression	Tetradecanoylph-orbol Acetate	D013755	Increases expression
Ethyl methanesulfonate	D005020	Decreases expression	Tobacco smoke pollution	D014028	Decreases expression
Fenthion	D005284	Increases expression	Tretinoin	D014212	Decreases expression
Folic acid	D005492	Decreases expression	Trichostatin A	C012589	Decreases expression
Formaldehyde	D005557	Decreases expression	Triptonide	C084079	Decreases expression
Fulvestrant	D000077267	Increases expression	Valproic acid	D014635	Decreases expression
Ionomycin	D015759	Increases expression	Vehicle emissions	D001335	Decreases expression
Ivermectin	D007559	Decreases expression			

## DISCUSSION

Original Research Epigenetic changes are reversible, heritable processes that affect gene expression without altering the DNA nucleotide sequence [29]. Abnormal epigenetic changes are closely associated with many human diseases, including cancer. Abnormal epigenetic pathways occur in the early stages of tumorigenesis and are therefore increasingly recognized as hallmarks of tumorigenesis [30]. Aberrant DNA methylation is currently the most widely studied epigenetic alteration in cancer. DNA methylation modifications are dynamically regulated by enzymes involved in modification, including DNA methylases and DNA demethylases, the so-called writers and erasers. In eukaryotes, methylation at the fifth position of cytosine to form 5-methylcytosine (5mC) is the most common DNA methylation modification; in vertebrate somatic cells, DNA 5mC modifications have been detected in more than 70% of CpG islands [31] and are often associated with transcriptional repression by transposable elements [32]. Recent studies have shown that another DNA methylation modification, namely the 6mA modification, which is widely present in the genome of prokaryotes and involved in the regulation of DNA replication, repair, transcription and other functions [33], is also widely present in eukaryotic genomes, including the human genome [3, 34–36]. The biological role of this ubiquitous and novel form of DNA methylation in human cancer is largely unknown.

N6AMT1 is the only writer so far of DNA 6mA methylation. It has a potentially huge role in the development of cancer, but little is known about its role in human cancer. This study focuses on the potential biological roles of N6AMT1 in 33 human tumor types. First, we investigated the subcellular localization of N6AMT1 protein and found that it was mainly localized in the nucleus, consistent with the functional properties of N6AMT1 as a DNA methylase. Generally, information at the protein level more directly reflects the biological effects of a gene; however, as there is a lack of public databases related to protein quantification, it is impossible to perform a comprehensive analysis at the protein level. Therefore, in this study, we carried out a comprehensive pan-cancer exploration of N6AMT1 at the transcriptome level, including early diagnosis, prognostic value, and immunological role.

We analyzed differences in the expression levels of N6AMT1 among the 33 tumor types. The results showed that it was significantly overexpressed in CHOL, COAD, LIHC and STAD. Consistent with previous studies, Lin et al. found that N6AMT1 was highly expressed in LIHC tissues, where it promoted proliferation, migration and invasion of LIHC cells and inhibited apoptosis [12]. We also investigated the correlations between N6AMT1 and various clinical parameters. The expression of N6AMT1 was higher in elderly patients with THYM and lower in male patients with SARC and UVM. These results may have important implications for guiding the selection of immunotherapy regimens for patients in different age and gender groups. Furthermore, the expression of N6AMT1 was significantly different at different stages in LUAD and THCA, implying that N6AMT1 may influence the progression of LUAD and THCA.

Collectively, these findings confirm the differential expression of N6AMT1 in a variety of cancers, suggesting a promising future for N6AMT1 in cancer diagnosis. It is worth noting that the search for early diagnostic markers of cancer has essential clinical significance, which can detect tumors as early as possible and greatly improve the clinical prognosis of patients. Therefore, we explored the diagnostic value of N6AMT1 in pan-cancer. The results showed that N6AMT1 showed excellent diagnostic value in multiple cancer types, especially in CHOL (AUC = 0.975) and LIHC (AUC = 0.882). In addition, we explored the prognostic value of N6AMT1 in different cancers by univariate Cox regression analysis and found that upregulation of N6AMT1 expression was associated with poor prognosis in CESC, LIHC, PCPG and PRAD. However, high expression of N6AMT1 was associated with better prognosis in PAAD and STAD. These results suggest that N6AMT1 may have different roles in different tumors. Taken together, these results clearly demonstrate that N6AMT1 is a potential novel diagnostic and prognostic marker in multiple cancer types. Excitingly, we found that N6AMT1 showed amazing diagnostic value and prognostic value in LIHC at the same time, which may mean that N6AMT1 has extremely high research value in LIHC.

In the past few years, immune checkpoint inhibitors (ICIs) targeting immune checkpoints have emerged as promising cancer treatments [37–39]. Future cancer treatment strategies are likely to aim at increasing the efficacy of ICIs. Owing to the plasticity of epigenetics, the development of drugs targeting epigenetic modifications has also been the subject of attention. DNA methyltransferase inhibitors and histone deacetylase inhibitors are clinical drugs currently in use that mainly target epigenetic modifications [40, 41]. Accumulating evidence suggests that tumor cells evade chemotherapy and host immune surveillance in general through epigenetic processes [42]. Studies have shown that epigenetic drugs can effectively reverse the immune evasion of tumor cells, for instance, by promoting tumor-associated neoantigen expression, improving immune cell recognition and regulating immune cell function in the TME [29, 43, 44]. Therefore, an important potential application of epigenetic drugs is their use in combination with ICIs to enhance clinical benefit in cancer patients compared with ICIs alone. Another important finding of this study was the potential value of N6AMT1 in cancer immunotherapy. We first investigated the correlation between N6AMT1 and TME. On the one hand, N6AMT1 was negatively correlated with both Immunescore and Stromalscore in LGG and MESO, with Stromalscore in SARC and with Immunescore in TGCT and THYM. On the other hand, N6AMT1 was negatively correlated with macrophages M0 in DLBC, positively correlated with macrophages M0 in UCS, positively correlated with macrophages M1 in THYM and negatively correlated with regulatory T cells. Macrophages M1 often indicate a pro-inflammatory phenotype and have anti-tumor associations [45, 46], whereas regulatory T cells often indicate an anti-inflammatory phenotype and have tumor-promoting associations [47]; both can be used as markers to guide cancer immunotherapy [47–49]. Therefore, N6AMT1 may represent a new target for immunotherapy in THYM patients.

Overall, N6AMT1 is negatively correlated with immune infiltration in a variety of cancers and inhibition of N6AMT1 expression may help to improve immune cell infiltration in cancer patients. In our study of immunosuppressants, immune activators and MHC molecules, most of the modulator-related molecules were inversely correlated with N6AMT1 (except in GBM and HNSC). These results support the development of drug combinations targeting N6AMT1 and modulator molecules. In addition, the GO and signal pathways related to N6AMT1 differed greatly in different tumors, but most were involved in cancer progression and immune-related directions, suggesting that N6AMT1 may affect cancer progression via its influence on the TME.

In this study, the associations of N6AMT1 with PDL1, TMB, MSI and MMR were also explored. PDL1, TMB, MSI and MMR are currently considered meaningful biomarkers for predicting ICI response. PDL1 expression is associated with response to ICI in a variety of cancers, including non-small-cell lung cancer, advanced gastric cancer and urothelial cancer [50–52]. Likewise, MMR systems have shown clinical benefit in immune checkpoint blockade in multiple cancer types [53]. MMR is an important DNA repair pathway with a key role in maintaining the fidelity of DNA replication and defects of MMR (dMMR) lead to MSI [54]. A recent study showed that most tumors with MSI-high/dMMR status exhibited high TMB [55]. A plausible explanation for this is that MSI-high/dMMR is associated with the occurrence of mutations. With the accumulation of mutations, TMB increases, which in turn leads to the formation of neoantigens and activates anti-tumor immune responses [56, 57]. Here, we investigated the association of N6AMT1 with PDL1, TMB and MSI in 33 tumor types. N6AMT1 expression was negatively correlated with PDL1, TMB and MSI in BRCA; with PDL1 and TMB in SARC; and with PDL1 and MSI in COAD. These results suggest that low expression of N6AMT1 may be conducive to the immunotherapy response in BRCA, SARC and COAD, especially BRCA. In addition, N6AMT1 and MMR-related genes were closely related in eight of the 33 cancer types studied; this provides some insight regarding the immunotherapeutic value of N6AMT1 in other tumors. Subsequently, we explored the correlations between N6AMT1 and immune responses in three immunotherapy cohorts. N6AMT1 was associated with a difference in treatment response only in the GSE168204 cohort representing metastatic melanoma, that is, N6AMT1 expression was lower in the nivolumab-responsive group of this cohort. These results were consistent with those of previous analyses. However, our study only explored three relevant cohorts, which made it difficult to fully describe the effect of N6AMT1 on immunotherapy response in cancer patients. Future studies should focus on basic and clinical studies of N6AMT1 in relation to immunotherapy in various cancers.

Finally, we explored chemicals related to N6AMT1. A total of 43 chemicals could affect N6AMT1 expression levels. Some well-known chemicals are included, including acetaminophen, cyclosporine, doxorubicin, and folic acid, which increase N6AMT1 expression, and sunitinib, tretinoin, and triptonide, which reduce N6AMT1 expression. These results are expected to provide some clinical guidance for cancer patients with abnormal N6AMT1 expression.

To date, there have been limited studies on N6AMT1 in cancer. This study is the first comprehensive analysis focusing on the role of N6AMT1 in 33 tumor types and its results demonstrate that N6AMT1 could be regarded as a potential target for cancer therapy. This study also provides a valuable basis for the diagnosis, prognosis and immunological roles of N6AMT1 in pan-cancer, especially in immunotherapy research provides some new insights.

However, this study still has some shortcomings. On the one hand, the main research results of this study come from the bioinformatics analysis of public databases, and the research on the biological function of N6AMT1 in specific cancers is lacking. Second, this study did not use a real-world cohort to validate the diagnostic and prognostic value of N6AMT1 in pan-cancer, as well as its predictive ability for immunotherapy. In the future, it is necessary to focus on the mechanism research and clinical drug development of N6AMT1 in specific cancers.

## CONCLUSIONS

This study is the first to explore the diagnostic, prognostic and immunotherapeutic value of N6AMT1 in pan-cancer. These results form part of a theoretical basis for further basic research and clinical experiments.
